# The F-coindex of some graph operations

**DOI:** 10.1186/s40064-016-1864-7

**Published:** 2016-02-29

**Authors:** Nilanjan De, Sk. Md. Abu Nayeem, Anita Pal

**Affiliations:** Department of Basic Sciences and Humanities (Mathematics), Calcutta Institute of Engineering and Management, Kolkata, West Bengal 700 040 India; Department of Mathematics, Aliah University, IIA/27, New Town, Kolkata, West Bengal 700 156 India; Department of Mathematics, National Institute of Technology, Durgapur, West Bengal India

**Keywords:** Topological index, Vertex degree, First and second Zagreb indices, F-index, F-coindex, Graph operations, Primary 05C35; Secondary 05C07, 05C40

## Abstract

The F-index of a graph is defined as the sum of cubes of the vertex degrees of the graph. In this paper, we introduce a new invariant which is named as F-coindex. Here, we study basic mathematical properties and the behavior of the newly introduced F-coindex under several graph operations such as union, join, Cartesian product, composition, tensor product, strong product, corona product, disjunction, symmetric difference of graphs and hence apply our results to find the F-coindex of different chemically interesting molecular graphs and nano-structures.

## Background

Topological indices are found to be very useful in chemistry, biochemistry and nanotechnology in isomer discrimination, structure–property relationship, structure-activity relationship and pharmaceutical drug design. Let *G* be a simple connected graph with vertex set *V*(*G*) and edge set *E*(*G*) respectively. Let, for any vertex ${v}\in V(G)$, ${{d}_{G}}(v)$ denotes its degree, that is the number of adjacent vertices of *v* in *G*. The complement of a graph *G* is denoted by $\bar{G}$ and is the simple graph with the same vertex set *V*(*G*) and any two vertices $uv\in E({\bar{G}})$ if and only if $uv\notin E(G)$. Thus $E(G)\cup E({\bar{G}})=E(K_n)$ and $|E(\bar{G})|=\frac{|V(G)|(|V(G)|-1)}{2}-|E(G)|$. Also the degree of a vertex *v* in $\bar{G}$ is given by ${{d}_{\bar{G}}}(v)=|V(G)|-1-{{d}_{G}}(v).$

The first and second Zagreb indices of a graph are among the most studied vertex-degree based topological indices. These indices were introduced by Gutman and Trinajstić (1972) to study the structure-dependency of the total $\pi$-electron energy ($\varepsilon$) and are denoted by $M_1(G)$ and $M_2(G)$ respectively. They are defined as

$${{M}_{1}}(G)=\sum \limits _{v\in V(G)}{{{d}_{G}}{{(v)}^{2}}}=\sum \limits _{uv\in E(G)}{[{{d}_{G}}(u)+{{d}_{G}}(v)]}$$

and

$${{M}_{2}}(G)=\sum \limits _{uv\in E(G)}{{{d}_{G}}(u){{d}_{G}}(v)}.$$

Another vertex-degree based topological index was defined in the same paper where the Zagreb indices were introduced, and that was shown to influence $\varepsilon$. This index was not further studied until it was studied by Furtula and Gutman (2015) in a recent article. They named this index as “forgotten topological index” or “F-index”. F-index of a graph *G* is denoted by *F*(*G*) and is defined as the sum of cubes of the vertex degrees of the graph.

$$\text{ i.e., }\;F(G)=\sum \limits _{v\in V(G)}{{{d}_{G}}{{(v)}^{3}}}.$$

It can be easily shown that the above definition is equivalent to

$$F(G)=\sum \limits _{uv\in E(G)}{\left[ {{d}_{G}}{{(u)}^{2}}+{{d}_{G}}{{(v)}^{2}}\right] }.$$

Very recently the present authors have studied the F-index of different graph operations in De et al. (2016).

Doslic (2008) introduced Zagreb coindices while computing weighted Wiener polynomial of certain composite graphs. In this case the sum runs over the edges of the complement of *G*. Thus the Zagreb coindices of *G* are defined as

$$\bar{{{M}}_{1}}(G)=\sum \limits _{uv\in E\left( \bar{G}\right) }{\left[ {{d}_{G}}(u)+{{d}_{G}}(v)\right] }$$

and

$$\bar{{{M}}_{2}}(G)=\sum \limits _{uv\in E\left( \bar{G}\right) }{{{d}_{G}}(u){{d}_{G}}(v)}.$$

Like Zagreb coindices, corresponding to F-index, we introduce here a new invariant, the F-coindex which is defined as follows.

$$\bar{F}({G})=\sum \limits _{uv\in E\left( \bar{G}\right) }{\left[ {{d}_{G}}{{(u)}^{2}}+{{d}_{G}}{{(v)}^{2}}\right] }.$$

Like Zagreb coindices, F-coindex of *G* is not the F-index of $\bar{G}$. Here the sum runs over $E(\bar{G})$, but the degrees are with respect to *G*.

## Motivation

According to the *International Academy of Mathematical Chemistry*, to identify whether any topological index is useful for prediction of chemical properties, the coorelation between the values of that topological index for different octane isomers and parameter values related to certain physicochemical property of them should be considered. Generally octane isomers are convenient for such studies, because the number of the structural isomers of octane is large (18) enough to make the statistical conclusion reliable. Furtula and Gutman (2015) showed that for octane isomers both $M_1$ and *F* yield correlation coefficient greater than 0.95 in case of entropy and acentric factor. They also improved the predictive ability of these index by considering a simple linear model in the form $({M_1}+{\lambda }F)$, where $\lambda$ varies from −20 to 20.

**Fig. 1:**
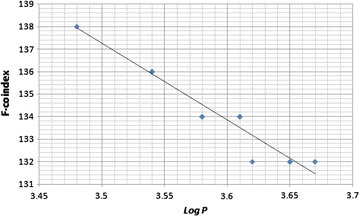
Experimental values of $\log P$ versus calculated values of *F*-coindices of octane isomers

In this paper, we find the correlation between the logarithm of the octanol-water partition coefficient (*P*) and the corresponding F-coindex values of octane isomers. The dataset of octane isomers (first three columns of Table 1) are taken from www.moleculardescriptors.eu/dataset/dataset.htm and the last two columns of Table 1 are computed from the definitions of *F*(*G*) and $\bar{F}(G)$. F-coindex values against $\log P$ values are plotted in Fig. 1. Here we find that the correlation coefficient between $\log P$ and $\bar{F}$ is 0.966, whereas the correlation coefficient between $\log P$ and $M_1$ and that between $\log P$ and *F* are 0.077 and 0.065 respectively. Thus using this F-coindex, we can predict the $\log P$ values with high accuracy.

**Table 1:** Experimental values of the logarithm of the octanol–water partition coefficient and the corresponding values of different topological indices of octane isomers

Molecules	Log *P*	$M_1(G)$	*F*(*G*)	$\bar{F}(G)$
Octane	3.67	26	50	132
2-Methyl-heptane	3.61	28	62	134
3-Methyl-heptane	3.61	28	62	134
4-Methyl-heptane	3.61	28	62	134
3-Ethyl-hexane	3.61	28	62	134
2,2-Dimethyl-hexane	3.65	32	92	132
2,3-Dimethyl-hexane	3.54	30	74	136
2,4-Dimethyl-hexane	3.54	30	74	136
2,5-Dimethyl-hexane	3.54	30	74	136
3,3-Dimethyl-hexane	3.65	32	92	132
3,4-Dimethyl-hexane	3.54	30	74	136
2-Methyl-3-ethyl-pentane	3.54	30	74	136
3-Methyl-3-ethyl-pentane	3.65	32	92	132
2,2,3-Trimethyl-pentane	3.58	34	104	134
2,2,4-Trimethyl-pentane	3.58	34	104	134
2,3,3-Trimethyl-pentane	3.58	34	104	134
2,3,4-Trimethyl-pentane	3.48	32	86	138
2,2,3,3-Tetramethyl-butane	3.62	38	134	132

Graph operations play an important role in chemical graph theory. Different chemically important graphs can be obtained by applying graph operations on some general or particular graphs. For example, the linear polynomial chain (or the ladder graph $L_n$) is the molecular graph related to the polynomial structure obtained by the Cartesian product of $P_2$ and $P_{n+1}$. The $C_4$ nanotube $TUC_4(m, n)$ is the Cartesian product of $P_n$ and $P_m$ and the $C_4$ nanotorus $TC_4(m, n)$ is the Cartesian product of $C_n$ and $C_m$. For a given graph *G*, one of the hydrogen suppressed molecular graph is the bottleneck graph, which is the corona product of $K_2$ and *G*. There are several studies on various topological indices under different graph operations available in the literature. Khalifeh et al. (2009) derived some exact formulae for computing first and second Zagreb indices under some graph operations. Das et al. (2013), derived some upper bounds for multiplicative Zagreb indices for different graph operations. Veylaki et al. (2015), computed third and hyper-Zagreb coindices of some graph operations. In De et al. (2014), the present authors computed some bounds and exact formulae of the connective eccentric index under different graph operations. Azari and Iranmanesh (2013) presented explicit formulas for computing the eccentric-distance sum of different graph operations. Interested readers are referred to Ashrafi et al. (2010), Khalifeh et al. (2008), Tavakoli et al. (2014), De et al. (2015a, b, c, d, Eskender and Vumar (2013) for other studies in this regard.

In this paper, we first derive some basic properties of F-coindex and hence present some exact expressions for the F-coindex of different graph operations such as union, join, Cartesian product, composition, tensor product, strong product, corona product, disjunction, symmetric difference of graphs. Also we apply our results to compute the F-coindex for some important classes of molecular graphs and nano-structures.

## Basic properties of F-coindex

From definition, the F-coindex for some special graphs such as complete graph, empty graph, path, cycle and complete bipartite graph on *n* vertices can be easily obtained as follows.

(i) $$\bar{F}\left( {{K}_{n}}\right) =\bar{F}\left( {{\bar{K}}_{n}}\right) =0$$,
(ii) $$\bar{F}\left( {{C}_{n}}\right) =4n(n-3)$$,
(iii) $$\bar{F}\left( {{P}_{n}}\right) =4{{n}^{2}}-18n+20$$,
(iv) $$\bar{F}({{K}_{m,n}})=mn(2mn-m-n).$$

Let for the graph *G* we use the notation $|V(G)|=n$ and ${|{E}(G)|}=m$. Also let ${|{E}(\bar{G})|}=\bar{m}$. Now first we explore some basic properties of F-coindex.

### **Proposition 1**

*LetGbe a simple graph withnvertices andmedges, then*

$$F\left( \bar{G}\right) =2{{(n-1)}^{2}}\left( \bar{m}-2m\right) +3(n-1){{M}_{1}}(G)-F(G).$$

### *Proof*

From definition of F-index, we have

$$\begin{aligned} F\left( \bar{G}\right)&= {} \sum \limits _{v\in V\left( \bar{G}\right) }{{{d}_{{\bar{G}}}}{{(v)}^{3}}}\\&= {} \sum \limits _{v\in V(G)}{{{\left[ n-1-{{d}_{G}}(v)\right] }^{3}}}\\&= \sum \limits _{v\in V(G)}{\left[ {{(n-1)}^{3}}-3{{(n-1)}^{2}}{{d}_{G}}(v)+3(n-1){{d}_{G}}{{(v)}^{2}}-{{d}_{G}}{{(v)}^{3}}\right] }\\&= {} n{{(n-1)}^{3}}-6m{{(n-1)}^{2}}+3(n-1){{M}_{1}}(G)-F(G)\\&= {} 2{{(n-1)}^{2}}\left( \bar{m}-2m\right) +3(n-1){{M}_{1}}(G)-F(G). \end{aligned}$$

$$\square$$

### **Proposition 2**

*LetGbe a simple graph withnvertices andmedges, then*

$$\bar{F}(G)=F\left( \bar{G}\right) -2(n-1){{M}_{1}}\left( \bar{G}\right) +2\bar{m}{{(n-1)}^{2}}.$$

### *Proof*

From definition of F-coindex, we have

$$\begin{aligned} \bar{F}(G)&= {} \sum \limits _{uv\notin E(G)}{\left[ {{d}_{G}}{{(u)}^{2}}+{{d}_{G}}{{(v)}^{2}}\right] }\\&= \sum \limits _{uv\in E\left( \bar{G}\right) }{\left[ {{\left\{ n-1-{{d}_{{\bar{G}}}}(u)\right\} }^{2}}+{{\left\{ n-1-{{d}_{{\bar{G}}}}(v)\right\} }^{2}}\right] }\\&= {} \sum \limits _{uv\in E\left( \bar{G}\right) }{\left[ {{(n-1)}^{2}}+{{d}_{{\bar{G}}}}{{(u)}^{2}}-2(n-1){{d}_{{\bar{G}}}}(u)+{{(n-1)}^{2}}+{{d}_{{\bar{G}}}}{{(v)}^{2}}-2(n-1){{d}_{{\bar{G}}}}(v)\right] }\\&= {} 2\bar{m}{{(n-1)}^{2}}+\sum \limits _{uv\in E\left( \bar{G}\right) }{\left[ {{d}_{{\bar{G}}}}{{(u)}^{2}}+{{d}_{{\bar{G}}}}{{(v)}^{2}}\right] }-2(n-1)\sum \limits _{uv\in E\left( \bar{G}\right) }{\left[ {{d}_{{\bar{G}}}}(u)+{{d}_{{\bar{G}}}}(v)\right] }\\&= {} 2\bar{m}{{(n-1)}^{2}}+F\left( \bar{G}\right) -2(n-1){{M}_{1}}\left( \bar{G}\right) . \end{aligned}$$

$$\square$$

An alternative expression for $\bar{F}(G)$ can be obtained by considering sum over the edges of *G* and $\bar{G}$ respectively as follows.

### **Proposition 3**

*LetGbe a simple graph with nvertices and m edges, then*

$$\bar{F}(G)=(n-1){{M}_{1}}(G)-F(G).$$

### *Proof*

From definition of F-index and F-coindex, it follows that

$$\begin{aligned} F(G)+\bar{F}(G)&= {} \sum \limits _{uv\in E(G)}{\left[ {{d}_{G}}{{(u)}^{2}}+{{d}_{G}}{{(v)}^{2}}\right] }+\sum \limits _{uv\notin E(G)}{\left[ {{d}_{G}}{{(u)}^{2}}+{{d}_{G}}{{(v)}^{2}}\right] }\\&= {} \sum \limits _{u,v\in V(G)}{\left[ {{d}_{G}}{{(u)}^{2}}+{{d}_{G}}{{(v)}^{2}}\right] }\\&= {} (n-1)\sum \limits _{v\in V(G)}{{{d}_{G}}{{(v)}^{2}}}=(n-1){{M}_{1}}(G), \end{aligned}$$

from where the desired result follows. $\square$

### **Proposition 4**

*Let G be a simple graph withnvertices and m edges, then*

$$\bar{F}\left( \bar{G}\right) =2m{{(n-1)}^{2}}-(n-1){{M}_{1}}(G)-\bar{F}(G).$$

### *Proof*

From definition of F-coindex, we have

$$\begin{aligned} \bar{F}\left( \bar{G}\right)&= {} \sum \limits _{uv\notin E\left( \bar{G}\right) }{\left[ {{d}_{{\bar{G}}}}{{(u)}^{2}}+{{d}_{{\bar{G}}}}{{(v)}^{2}}\right] }\\&= {} \sum \limits _{uv\in E(G)}{\left[ {{\left\{ n-1-{{d}_{G}}(u)\right\} }^{2}}+{{\left\{ n-1-{{d}_{G}}(v)\right\} }^{2}}\right] }\\&= {} \sum \limits _{uv\in E(G)}{\left[ {{(n-1)}^{2}}+{{d}_{G}}{{(u)}^{2}}-2(n-1){{d}_{G}}(u)+{{(n-1)}^{2}}+{{d}_{G}}{{(v)}^{2}}-2(n-1){{d}_{G}}(v)\right] }\\&= {} 2m{{(n-1)}^{2}}+\sum \limits _{uv\in E(G)}{\left[ {{d}_{G}}{{(u)}^{2}}+{{d}_{G}}{{(v)}^{2}}\right] }-2(n-1)\sum \limits _{uv\in E(G)}{\left[ {{d}_{G}}(u)+{{d}_{G}}(v)\right] }\\&= {} 2m{{(n-1)}^{2}}+F(G)-2(n-1){{M}_{1}}(G)\\&= {} 2m{{(n-1)}^{2}}-(n-1){{M}_{1}}(G)-\bar{F}(G). \end{aligned}$$

$$\square$$

## Main results

In the following, we study F-coindex of various graph operations like union, join, Cartesian product, composition, tensor product, strong product, corona product, disjunction, symmetric difference of graphs. These operations are binary and if not indicated otherwise, we use the notation $V(G_i)$ for the vertex set, ${{E}(G_i)}$ for the edge set, ${{n}_{i}}$ for the number of vertices and ${{m}_{i}}$ for the number of edges of the graph ${{G}_{i}}$ respectively. Also let ${\bar{{m}}_{i}}$ denote the number of edges of the graph ${{\bar{G}}_{i}}.$

### Union

The union of two graphs ${{G}_{1}}$ and ${{G}_{2}}$ is the graph denoted by ${{G}_{1}}\cup {{G}_{2}}$ with the vertex set $V({{G}_{1}})\cup V({{G}_{2}})$ and edge set $E({{G}_{1}})\cup E({{G}_{2}})$. In this case we assume that $V({{G}_{1}})$ and $V({{G}_{2}})$ are disjoint. The degree of a vertex *v* of ${{G}_{1}}\cup {{G}_{2}}$ is equal to that of the vertex in the component ${{G}_{i}}\,(i=1,2)$ which contains it. In the following preposition we calculate the F-coindex of ${{G}_{1}}\cup {{G}_{2}}.$

#### **Proposition 5**

*Let G be a simple graph withnvertices and m edges, then*

$$\bar{F}\left( {{G}_{1}}\cup {{G}_{2}}\right) =\bar{F}\left( {{G}_{1}}\right) +\bar{F}\left( {{G}_{2}}\right) +{{n}_{2}}{{M}_{1}}\left( {{G}_{1}}\right) +{{n}_{1}}{{M}_{1}}\left( {{G}_{2}}\right).$$

#### *Proof*

From definition of F-coindex, it is clear that, the F-coindex of ${{G}_{1}}\cup {{G}_{2}}$ is equal to the sum of the F-coindices of the components ${{G}_{i}}(i=1,2)$, in addition to the contributions of the missing edges between the components which form the edge set of the complete bipartite graph ${{K}_{{{n}_{1}},{{n}_{2}}}}$. The contribution of these missing edges is given by

$$\sum \limits _{u\in V\left( {{G}_{1}}\right) }{\left[ \sum \limits _{v\in V\left( {{G}_{2}}\right) }{\left\{ {{d}_{G}}{{(u)}^{2}}+{{d}_{G}}{{(v)}^{2}}\right\} }\right] ={{n}_{2}}{{M}_{1}}\left( {{G}_{1}}\right) +{{n}_{1}}{{M}_{1}}\left( {{G}_{2}}\right)},$$

from where the desired result follows. $\square$

### Join

The join of two graphs ${{G}_{1}}$ and ${{G}_{2}}$ with disjoint vertex sets $V({{G}_{1}})$ and $V({{G}_{2}})$ is the graph denoted by ${{G}_{1}}+{{G}_{2}}$ with the vertex set $V({{G}_{1}})\cup V({{G}_{2}})$ and edge set $E({{G}_{1}})\cup E({{G}_{2}})\cup \{uv:u\in V({{G}_{1}}),v\in V({{G}_{2}})\}$. Thus in the sum of two graphs, all the vertices of one graph is connected with all the vertices of the other graph, keeping all the edges of both graphs. Thus the degree of the vertices of ${{G}_{1}}+{{G}_{2}}$ is given by

$$\begin{aligned} {{d}_{{{G}_{1}}+{{G}_{2}}}}(v) = \left\{ \begin{array}{ll} {{d}_{{{G}_{1}}}}(v)+{{n}_{2}},&{} \quad v\in V({{G}_{1}})\\ {{d}_{{{G}_{2}}}}(v)+{{n}_{1}},&{}\quad v\in V({{G}_{2}}). \end{array}\right. \end{aligned}$$

In the following proposition the F-coindex of ${{G}_{1}}+{{G}_{2}}$ is calculated.

#### **Proposition 6**

*Let Gbe a simple graph with nvertices and m edges, then*

$$\bar{F}({{G}_{1}}+{{G}_{2}})=\bar{F}({{G}_{1}})+\bar{F}({{G}_{2}})+2{{n}_{2}}{{\bar{M}}_{1}}({{G}_{1}})+2{{n}_{1}}{{\bar{M}}_{1}}({{G}_{2}})+2{{n}_{2}}^{2}{{\bar{m}}_{1}}+2{{n}_{1}}^{2}{{\bar{m}}_{2}}.$$

#### *Proof*

From definition of ${{G}_{1}}+{{G}_{2}}$, it is clear that the contribution of the edges connecting the vertices of ${{G}_{1}}$ with those of ${{G}_{2}}$ is zero. So the F-coindex of ${{G}_{1}}+{{G}_{2}}$ is given by

$$\begin{aligned} \bar{F}({{G}_{1}}+{{G}_{2}})&= {} \sum \limits _{uv\notin E({{G}_{1}}+{{G}_{2}})}{\left[ {{d}_{({{G}_{1}}+{{G}_{2}})}}{{(u)}^{2}}+{{d}_{({{G}_{1}}+{{G}_{2}})}}{{(v)}^{2}}\right] }\\&= {} \sum \limits _{uv\notin E({{G}_{1}})}{\left[ {{d}_{({{G}_{1}}+{{G}_{2}})}}{{(u)}^{2}}+{{d}_{({{G}_{1}}+{{G}_{2}})}}{{(v)}^{2}}\right] }\\&\quad +\,\sum \limits _{uv\notin E({{G}_{2}})}{\left[ {{d}_{({{G}_{1}}+{{G}_{2}})}}{{(u)}^{2}}+{{d}_{({{G}_{1}}+{{G}_{2}})}}{{(v)}^{2}}\right] } ={{J}_{1}}+{{J}_{2}}. \end{aligned}$$

Now,

$$\begin{aligned} {{J}_{1}}&= {} \sum \limits _{uv\notin E({{G}_{1}})}{\left[ {{d}_{({{G}_{1}}+{{G}_{2}})}}{{(u)}^{2}}+{{d}_{({{G}_{1}}+{{G}_{2}})}}{{(v)}^{2}}\right] }\\&= {} \sum \limits _{uv\notin E({{G}_{1}})}{\left[ {{({{d}_{{{G}_{1}}}}(u)+{{n}_{2}})}^{2}}+{{({{d}_{{{G}_{1}}}}(v)+{{n}_{2}})}^{2}}\right] }\\&= {} \sum \limits _{uv\notin E({{G}_{1}})}{\left[ {{d}_{{{G}_{1}}}}{{(u)}^{2}}+{{n}_{2}}^{2}+2{{n}_{2}}{{d}_{{{G}_{1}}}}(u)+{{d}_{{{G}_{1}}}}{{(v)}^{2}}+{{n}_{2}}^{2}+2{{n}_{2}}{{d}_{{{G}_{1}}}}(v)\right] }\\&= {} \sum \limits _{uv\notin E({{G}_{1}})}{\left[ {{d}_{{{G}_{1}}}}{{(u)}^{2}}+{{d}_{{{G}_{1}}}}{{(v)}^{2}}\right] }+2{{n}_{2}}\sum \limits _{uv\notin E({{G}_{1}})}{\left[ {{d}_{{{G}_{1}}}}(u)+{{d}_{{{G}_{1}}}}(v)\right] }+2{{n}_{2}}^{2}{{\bar{m}}_{1}}\\&= {} \bar{F}({{G}_{1}})+2{{n}_{2}}{{\bar{M}}_{1}}({{G}_{1}})+2{{n}_{2}}^{2}{{\bar{m}}_{1}}. \end{aligned}$$

Similarly, we get

$${{J}_{2}}=\bar{F}({{G}_{2}})+2{{n}_{1}}{{\bar{M}}_{1}}({{G}_{2}})+2{{n}_{1}}^{2}{{\bar{m}}_{2}}.$$

Combining ${{J}_{1}}$ and ${{J}_{2}}$ we get the desired result after simplification. $\square$

#### *Example 1*

The complete bipartite graph ${{K}_{p,q}}$ can be defined as ${{K}_{p,q}}={{\bar{K}}_{p}}+{{\bar{K}}_{q}}$. So its F-coindex can be calculated from the previous proposition as $\bar{F}({{K}_{p,q}})=pq(2pq-p-q).$

The suspension of a graph *G* is defined as sum of *G* with a single vertex. So from the previous proposition the following corollary follows.

#### **Corollary 1**

*The F-coindex of suspension of G is given by*

$$\bar{F}(G+K_1)=\bar{F}(G)+2{{\bar{M}}_{1}}(G)+2\bar{m}.$$

#### *Example 2*

The star graph ${{S}_{n}}$ with *n* vertices is the suspension of empty graph ${{\bar{K}}_{n-1}}$. So its F-coindex can be calculated from the previous corollary as $\bar{F}({{S}_{n}})=(n-1)(n-2).$

#### *Example 3*

The wheel graph ${{W}_{n}}$ on $(n+1)$ vertices is the suspension of ${{C}_{n}}$. So from the previous corollary its F-coindex is given by $\bar{F}({{W}_{n}})=9n(n-3).$

#### *Example 4*

The fan graph ${{F}_{n}}$ on $(n+1)$ vertices is the suspension of ${{P}_{n}}$. So from the previous corollary its F-coindex is given by $\bar{F}({{W}_{n}})=9{{n}^{2}}-37n+38.$

We now extend the join operation to more than two graphs. Let $$G_1,$$$$G_2,\ldots ,G_k$$ be *k* graphs. Then, the degree of a vertex *v* in $${{G}_{1}}+{{G}_{2}}+\cdots +{{G}_{k}}$$ is given by $${{d}_{{{G}_{1}}+{{G}_{2}}+\cdots +{G}_{k}}(v) ={{d}_{{{G}_{i}}}}(v)+{n}-{{n}_{i}}}$$, where *v* is originally a vertex of the graph $$G_i$$ and $$n={{n}_{1}}+{{n}_{2}}+\cdots +{{n}_{k}}$$. Also let $${\bar{n}_i}=n-{n_i}$$.

#### **Proposition 7**

*The F-coindex of*${{G}_{1}}+{{G}_{2}}+\cdots +{{G}_{k}}$*is given by*

$$\bar{F}\left( {{G}_{1}}+{{G}_{2}}+\cdots +{{G}_{k}}\right) =\sum \limits _{i=1}^{k}{\bar{F}({{G}_{i}})}+2\sum \limits _{i=1}^{k}{{{{\bar{n}}}_{i}}{{{\bar{M}}}_{1}}({{G}_{i}})}+2\sum \limits _{i=1}^{k}{{{{\bar{n}}}_{i}}^{2}{{{\bar{m}}}_{i}}}.$$

#### *Proof*

We have from definition of F-coindex

$$\begin{aligned}\bar{F}\left( {{G}_{1}}+{{G}_{2}}+\cdots +{{G}_{k}}\right)&= {} \sum \limits _{i=1}^{k}{\sum \limits _{uv\notin E({{G}_{i}})}{\left[ {{d}_{\left( {{G}_{1}}+{{G}_{2}}+\cdots +{{G}_{k}}\right) }}{{(u)}^{2}}+{{d}_{\left( {{G}_{1}}+{{G}_{2}}+\cdots +{{G}_{k}}\right) }}{{(v)}^{2}}\right] }}\\&= {} \sum \limits _{i=1}^{k}{\sum \limits _{uv\notin E({{G}_{1}})}{\left[ {{\left( {{d}_{{{G}_{i}}}}(u)+{{{\bar{n}}}_{i}}\right) }^{2}}+{{\left( {{d}_{{{G}_{i}}}}(v)+{{{\bar{n}}}_{i}}\right) }^{2}}\right] }}\\&= {} \sum \limits _{i=1}^{k}{\sum \limits _{uv\notin E({{G}_{1}})}{\left[ {{d}_{{{G}_{i}}}}{{(u)}^{2}}+{{{\bar{n}}}_{i}}^{2}+2{{{\bar{n}}}_{i}}{{d}_{{{G}_{i}}}}(u)+{{d}_{{{G}_{i}}}}{{(v)}^{2}}+{{{\bar{n}}}_{i}}^{2}+2{{{\bar{n}}}_{i}}{{d}_{{{G}_{i}}}}(v)\right] }}\\&= {} \sum \limits _{i=1}^{k}{\sum \limits _{uv\notin E({{G}_{1}})}{\left[ {{d}_{{{G}_{i}}}}{{(u)}^{2}}+{{d}_{{{G}_{i}}}}{{(v)}^{2}}\right] }}\\&\quad +\,\sum \limits _{i=1}^{k}{2{{{\bar{n}}}_{i}}\sum \limits _{uv\notin E({{G}_{1}})}{\left[ {{d}_{{{G}_{i}}}}(u)+{{d}_{{{G}_{i}}}}(v)\right] }}+\sum \limits _{i=1}^{k}{2{{{\bar{n}}}_{i}}^{2}{{{\bar{m}}}_{i}}}\\&= {} \sum \limits _{i=1}^{k}{\bar{F}({{G}_{i}})}+2\sum \limits _{i=1}^{k}{{{{\bar{n}}}_{i}}{{{\bar{M}}}_{1}}({{G}_{i}})}+2\sum \limits _{i=1}^{k}{{{{\bar{n}}}_{i}}^{2}{{{\bar{m}}}_{i}}}, \end{aligned}$$

which completes the proof. $\square$

### Cartesian product

The Cartesian product of $G_1$ and $G_2$, denoted by $G_1\times G_2$, is the graph with vertex set $V(G_1)\times V(G_2)$ and any two vertices $({{u}_{p}},{{v}_{r}})$ and $({{u}_{q}},{{v}_{s}})$ are adjacent if and only if [${{u}_{p}}={{u}_{q}}\in V(G_1)$ and ${{v}_{r}}{{v}_{s}}\in E(G_2)$] or [${{v}_{r}}={{v}_{s}}\in V(G_2)$ and ${{u}_{p}}{{u}_{q}}\in E(G_1)$]. Thus we have, ${{d}_{{{G}_{1}}\times {{G}_{2}}}}(a,b)={{d}_{{{G}_{1}}}}(a)+{{d}_{{{G}_{2}}}}(b)$. In the following preposition we calculate the F-coindex of ${{G}_{1}}\times {{G}_{2}}$.

#### **Proposition 8**

*The F-coindex of*${{G}_{1}}\times {{G}_{2}}$*is given by*

$$\begin{aligned} \bar{F}({{G}_{1}}\times {{G}_{2}})&= {} \left\{ {{n}_{2}}({{n}_{1}}{{n}_{2}}-1)-6{{m}_{2}}\right\} {{M}_{1}}({{G}_{1}})+\{{{n}_{1}}({{n}_{1}}{{n}_{2}}-1)\\&\quad -6{{m}_{1}}\}{{M}_{1}}({{G}_{2}})-{{n}_{2}}F({{G}_{1}})-{{n}_{1}}F({{G}_{2}})+8{{m}_{1}}{{m}_{2}}({{n}_{1}}{{n}_{2}}-1).\\ \end{aligned}$$

#### *Proof*

Applying Theorem 1 of Khalifeh et al. (2009) and Theorem 3 of De et al. in Proposition 3 we get

$$\begin{aligned} \bar{F}({{G}_{1}}\times {{G}_{2}})&= {} \left( |V({{G}_{1}}\times {{G}_{2}})|-1\right) {{M}_{1}}({{G}_{1}}\times {{G}_{2}})-F({{G}_{1}}\times {{G}_{2}})\\&= {} ({{n}_{1}}{{n}_{2}}-1)\left[ {{n}_{2}}{{M}_{1}}({{G}_{1}})+{{n}_{1}}{{M}_{1}}({{G}_{2}})+8{{m}_{1}}{{m}_{2}}\right] -\left. [{{n}_{2}}F({{G}_{1}})+{{n}_{1}}F({{G}_{2}})\right. \\&\left. \quad +\,6{{m}_{2}}{{M}_{1}}({{G}_{1}})+6{{m}_{1}}{{M}_{1}}({{G}_{2}})\right] \\&= {} ({{n}_{1}}{{n}_{2}}-1){{n}_{2}}{{M}_{1}}({{G}_{1}})+({{n}_{1}}{{n}_{2}}-1){{n}_{1}}{{M}_{1}}({{G}_{2}})+8{{m}_{1}}{{m}_{2}}({{n}_{1}}{{n}_{2}}-1)\\&\quad -\,{{n}_{2}}F({{G}_{1}})-{{n}_{1}}F({{G}_{2}})-6{{m}_{2}}{{M}_{1}}({{G}_{1}})-6{{m}_{1}}{{M}_{1}}({{G}_{2}}), \end{aligned}$$

from where the desired result follows after simplification. $\square$

#### *Example 5*

The Ladder graph $L_n$ (linear polynomial chain) is the Cartesian product of $P_2$ and $P_{n+1}$. Thus from the last proposition the following result follows

$$\bar{F}({{L}_{n}})=36{{n}^{2}}-40n+20.$$

#### *Example 6*

$TUC_4(m, n)$ and $TC_4(m,n)$ denote a $C_4$ nanotube and nanotorus respectively. Then $TUC_4(m, n)\cong {P_n}\times {C_m}$ and $TC_4(m,n)\cong {C_n}\times {C_m},$ and so $\bar{F}(TUC_4(m, n))=16{m^2}{n^2}-14{m^2}n-80mn+88m$ and $\bar{F}(TC_4(m,n))=16{m}{n}(mn-5)$.

### Composition

The composition of two graphs ${{G}_{1}}$ and ${{G}_{2}}$ is denoted by ${{G}_{1}}[{{G}_{2}}]$ and any two vertices $({{u}_{1}},{{u}_{2}})$ and $({{v}_{1}},{{v}_{2}})$ are adjacent if and only if ${{u}_{1}}{{v}_{1}}\in E({{G}_{1}})$ or [${{u}_{1}}={{v}_{1}}$ and ${{u}_{2}}{{v}_{2}}\in E({{G}_{2}})$]. The vertex set of ${{G}_{1}}[{{G}_{2}}]$ is $V({{G}_{1}})\times V({{G}_{2}})$ and the degree of a vertex (*a*, *b*) of ${{G}_{1}}[{{G}_{2}}]$ is given by ${{d}_{{{G}_{1}}[{{G}_{2}}]}}(a,b)={{n}_{2}}{{d}_{{{G}_{1}}}}(a)+{{d}_{{{G}_{2}}}}(b).$ In the following proposition we compute the F-coindex of the composition of two graphs.

#### **Proposition 9**

*The F-coindex of*${{G}_{1}}[{{G}_{2}}]$ is given by

$$\begin{aligned} \bar{F}({{G}_{1}}[{{G}_{2}}])&= {} {{n}_{2}}^{2}\left\{ {{n}_{2}}({{n}_{1}}{{n}_{2}}-1)-6{{m}_{2}}\right\} {{M}_{1}}({{G}_{1}})+\{{{n}_{1}}({{n}_{1}}{{n}_{2}}-1)-6{{n}_{2}}{{m}_{1}}\}{{M}_{1}}({{G}_{2}})\\&\quad-{{n}_{2}}^{4}F({{G}_{1}}) -\,{{n}_{1}}F({{G}_{2}})+8{{n}_{2}}{{m}_{1}}{{m}_{2}}({{n}_{1}}{{n}_{2}}-1).\\ \end{aligned}$$

The proof of the above proposition follows from the expressions of first Zagreb index and F-index of strong product graphs given in Theorems 3 and 4 of Khalifeh et al. (2009) and De et al. respectively.

#### *Example 7*

The fence graph is the composition of ${{P}_{n}}$ and ${{P}_{2}}$ and the Closed fence graph is the composition of ${{C}_{n}}$ and ${{P}_{2}}$. Thus, we have

(i) $$\bar{F}({{P}_{n}}[{{P}_{2}}])=100{{n}^{2}}-428n+456$$,
(ii) $$\bar{F}({{C}_{n}}[{{P}_{2}}])=100{{n}^{2}}-300n.$$

### Tensor product

The tensor product of two graphs ${{G}_{1}}$ and ${{G}_{2}}$ is denoted by ${{G}_{1}}\otimes {{G}_{2}}$ and any two vertices $({{u}_{1}},{{v}_{1}})$ and $({{u}_{2}},{{v}_{2}})$ are adjacent if and only if ${{u}_{1}}{{u}_{2}}\in E({{G}_{1}})$ and ${{v}_{1}}{{v}_{2}}\in E({{G}_{2}})$. The degree of a vertex (*a*, *b*) of ${{G}_{1}}\otimes {{G}_{2}}$ is given by ${{d}_{{{G}_{1}}\otimes {{G}_{2}}}}(a,b)={{d}_{{{G}_{1}}}}(a){{d}_{{{G}_{2}}}}(b)$. In the following proposition, the F-coindex of the tensor product of two graphs is computed.

#### **Proposition 10**

*The F-coindex of*${{G}_{1}}\otimes {{G}_{2}}$*is given by*

$$\bar{F}({{G}_{1}}\otimes {{G}_{2}})=({{n}_{1}}{{n}_{2}}-1){{M}_{1}}({{G}_{1}}){{M}_{1}}({{G}_{2}})-F({{G}_{1}})F({{G}_{2}}).$$

The proof follows from the expressions ${M_1}({{G}_{1}}\otimes {{G}_{2}})={{M}_{1}}({{G}_{1}}){{M}_{1}}({{G}_{2}})$ established in Theorem 2.1 of Yarahmadi (2011) and $F({{G}_{1}}\otimes {{G}_{2}})=F({{G}_{1}})F({{G}_{2}})$ established in Theorem 7 of De et al.

#### *Example 8*

(i) $$\bar{F}({{P}_{n}}\otimes {{P}_{m}})=4(mn-1)(2n-3)(2m-3)-4(4n-7)(4m-7)$$
(ii) $$\bar{F}({{C}_{n}}\otimes {{C}_{m}})=16mn(mn-5)$$
(iii) $$\bar{F}({{K}_{n}}\otimes {{K}_{m}})=nm{{(n-1)}^{2}}{{(m-1)}^{2}}(m+n-1)$$
(iv) $$\bar{F}({{P}_{n}}\otimes {{C}_{m}})=4m(mn-1)(2n-3)(2m-3)-4(4n-7)(4m-7)$$
(v) $$\bar{F}({{P}_{n}}\otimes {{k}_{m}})=m(mn-1)(4n-6){{(m-1)}^{2}}-m(8n-14){(m-1)^3}$$
(vi) $$\bar{F}({{C}_{n}}\otimes {{K}_{m}})=4nm{{(m-1)}^{2}}(mn-2m+1)$$.

### Strong product graphs

The strong product of two graphs $G_1$ and $G_2$ is denoted by ${{G}_{1}}\boxtimes {{G}_{2}}$. It has the vertex set $V(G_1)\times V(G_2)$ and any two vertices $({{u}_{p}},{{v}_{r}})$ and $({{u}_{q}},{{v}_{s}})$ are adjacent if and only if [${{u}_{p}}={{u}_{q}}\in V(G_1)$ and ${{v}_{r}}{{v}_{s}}\in E(G_2)$] or [${{v}_{r}}={{v}_{s}}\in V(G_2)$ and ${{u}_{p}}{{u}_{q}}\in E(G_1)$] or [${{u}_{p}}{{u}_{q}}\in E(G_1)$ and ${{v}_{r}}{{v}_{s}}\in E(G_2)$]. Note that if both $G_1$ and $G_2$ are connected then ${{G}_{1}}\boxtimes {{G}_{2}}$ is also connected. The degree of a vertex (*a*, *b*) of ${{G}_{1}}\boxtimes {{G}_{2}}$ is given by

$${{d}_{{{G}_{1}}\boxtimes {{G}_{2}}}}(a,b)={{d}_{{{G}_{1}}}}(a)+{{d}_{{{G}_{2}}}}(b)+{{d}_{{{G}_{1}}}}(a){{d}_{{{G}_{2}}}}(b).$$

In the following proposition we compute the F-coindex of the strong product of two graphs.

#### **Proposition 11**

*The F-coindex of*${{G}_{1}}\boxtimes {{G}_{2}}$*is given by*

$$\begin{aligned} \bar{F}({{G}_{1}}\boxtimes {{G}_{2}})&= {} \{({{n}_{1}}{{n}_{2}}-1)({{n}_{2}}+4{{m}_{2}})-6{{m}_{2}}\}{{M}_{1}}({{G}_{1}})+\{({{n}_{1}}{{n}_{2}}-1)({{n}_{1}}+4{{m}_{1}})-6{{m}_{1}}\}{{M}_{1}}({{G}_{2}})\\&\quad +\,({{n}_{1}}{{n}_{2}}-7){{M}_{1}}({{G}_{1}}){{M}_{1}}({{G}_{2}})-({{n}_{2}}+6{{m}_{2}})F({{G}_{2}})-3F({{G}_{2}}){{M}_{1}}({{G}_{1}})\\&\quad -\,3F({{G}_{1}}){{M}_{1}}({{G}_{2}})-F({{G}_{1}})F({{G}_{2}})-8{{m}_{1}}{{m}_{2}}({{n}_{1}}{{n}_{2}}-1).\\ \end{aligned}$$

The proof follows from the expressions of first Zagreb index and F-index of strong product graphs from Theorems 2.6 and 6 of Tavakoli et al. (2013) and De et al. respectively.

### Corona product

The corona product ${{G}_{1}}\circ {{G}_{2}}$ of two graphs $G_1$ and $G_2$ is obtained by taking one copy of ${{G}_{1}}$ and ${{n}_{1}}$ copies of ${{G}_{2}}$ and by joining each vertex of the *i*th copy of ${{G}_{2}}$ to the *i*th vertex of ${{G}_{1}}$, where $1\le i\le {{n}_{1}}$. The corona product of ${{G}_{1}}$ and ${{G}_{2}}$ has total $({{n}_{1}}{{n}_{2}}+{{n}_{1}})$ number of vertices and $({{m}_{1}}+{{n}_{1}}{{m}_{2}}+{{n}_{1}}{{n}_{2}})$ number of edges. Different topological indices under the corona product of graphs have already been studied (Yarahmadi and Ashrafi 2012; De et al. 2015e; Pattabiraman and Kandan 2014). It is easy to see that the degree of a vertex *v* of ${{G}_{1}}\circ {{G}_{2}}$ is given by

$$\begin{aligned} {{d}_{{{G}_{1}}\circ {{G}_{2}}}}(v) = \left\{ \begin{array}{ll} {{d}_{{{G}_{1}}}}(v)+{{n}_{2}},&{}\quad v\in V({{G}_{1}})\\ {{d}_{{{G}_{2,i}}}}(v)+1,&{}\quad v\in V({{G}_{2,i}}),\quad i=1,2,\ldots ,{n_1}. \end{array}\right. \end{aligned}$$

In the following proposition, the F-coindex of the corona product of two graphs is computed.

#### **Proposition 12**

*The F-coindex of*${{G}_{1}}\circ {{G}_{2}}$*is given by*

$$\begin{aligned} \bar{F}({{G}_{1}}\circ {{G}_{2}})&= {} ({{n}_{1}}{{n}_{2}}+{{n}_{1}}-3{{n}_{2}}-1){{M}_{1}}({{G}_{1}})+{{n}_{1}}({{n}_{1}}{{n}_{2}}+{{n}_{1}}-4){{M}_{1}}({{G}_{2}})\\&\quad -\,F({{G}_{1}})-{{n}_{1}}F({{G}_{2}}) +4({{n}_{1}}{{n}_{2}}+{{n}_{1}}-1)({{n}_{1}}{{m}_{2}}+{{n}_{2}}{{m}_{1}})+{{n}_{1}}{{n}_{2}}({{n}_{1}}{{n}_{2}}\\&\quad +\,{{n}_{1}}-1)({{n}_{2}}+1) -6{{n}_{1}}{{m}_{2}}-6{{n}_{2}}^{2}{{m}_{1}}-{{n}_{1}}{{n}_{2}}\left( {{n}_{2}}^{2}+1\right) .\\ \end{aligned}$$

The proof of the above proposition follows from the relations

$${{M}_{1}}({{G}_{1}}\circ {{G}_{2}})={{M}_{1}}({{G}_{1}})+{{n}_{1}}{{M}_{1}}({{G}_{2}})+4({{n}_{2}}{{m}_{1}}+{{n}_{1}}{{m}_{2}})+{{n}_{1}}{{n}_{2}}({{n}_{2}}+1)$$

given in Theorem 2.8 of Yarahmadi and Ashrafi (2012) and

$$\begin{aligned} F({{G}_{1}}\circ {{G}_{2}})&= {} F({{G}_{1}})+{{n}_{1}}F({{G}_{2}})+3{{n}_{2}}{{M}_{1}}({{G}_{1}})+3{{n}_{1}}{{M}_{1}}({{G}_{2}})\\&\quad +\,6{{n}_{2}}^{2}{{m}_{1}}+6{{n}_{1}}{{m}_{2}}+{{n}_{1}}{{n}_{2}}({{n}_{2}}^{2}+1) \end{aligned}$$

given in Theorem 7 of De et al.

#### *Example 9*

One of the hydrogen suppressed molecular graph is the bottleneck graph of a graph *G*, is the corona product of ${K}_{2}$ and *G*, where *G* is a given graph. F-coindex of bottleneck graph of *G* is given by

$$\bar{F}({K}_{2}\circ G)=2F(G)+6{{M}_{1}}(G)+2n^3+6n^2+8n+12m+2,$$

where *n* is the number of vertices of *G*.

A *t*-thorny graph is obtained by joining *t*-number of thorns (pendent edges) to each vertex of a given graph *G*. A variety of topological indices of thorn graphs have been studied by a number of researchers (De 2012a, b; Alizadeh et al. 2014). It is well known that, the *t*-thorny graph of *G* is defined as the corona product of *G* and complement of complete graph with *t* vertices $\bar{K_t}$. Thus from the previous theorem the following corollary follows.

#### **Corollary 2**

*The F-coindex of t-thorny graph of G is given by*

$$\begin{aligned} \bar{F}\left( {{G}^{t}}\right)&= (nt+n-3t-1){{M}_{1}}(G)-F(G)+4mt(nt+n-1)-6m{{t}^{2}}\\&\quad +nt(t+1)(nt+n-1)-nt({{t}^{2}}+1).\end{aligned}$$

#### *Example 10*

The F-coindex of *t*-thorny graph of ${{C}_{n}}$ and ${{P}_{n}}$ are given by

(i) $$\bar{F}\left( {{C}_{n}}^{t}\right) ={{n}^{2}}{{t}^{3}}-n{{t}^{3}}+6{{n}^{2}}{{t}^{2}}-7n{{t}^{2}}+9{{n}^{2}}t-18nt+4{{n}^{2}}-12n$$
(ii) $$\bar{F}\left( {{P}_{n}}^{t}\right) ={{n}^{2}}{{t}^{3}}-n{{t}^{3}}+6{{n}^{2}}{{t}^{2}}-11n{{t}^{2}}+9{{n}^{2}}t-28nt+4{{n}^{2}}+6{{t}^{2}}-18n+22t+20$$.

### Disjunction

The disjunction of two graphs ${{G}_{1}}$ and ${{G}_{2}}$, denoted by ${{G}_{1}}\wedge {{G}_{2}}$, consists of the vertex set $V(G_1)\times V(G_2)$ and two vertices $(u_1,v_1)$ and $(u_2,v_2)$ are adjacent whenever ${u_1}{u_2}\in {E(G_1)}$ or ${v_1}{v_2}\in {E(G_2)}$. Clearly, the degree of a vertex $(u_1,u_2)$ of ${{G}_{1}}\wedge {{G}_{2}}$ is given by

$${{d}_{{{G}_{1}}\wedge {{G}_{2}}}}(u_1,u_2)={n_1}{{d}_{{{G}_{1}}}}(u_1)+{n_2}{{d}_{{{G}_{2}}}}(u_2)-{{d}_{{{G}_{1}}}}(u_1){{d}_{{{G}_{2}}}}(u_2).$$

In the following theorem we obtain the F-coindex of the disjunction of two graphs.

#### **Proposition 13**

*The F-coindex of*${{G}_{1}}\wedge {{G}_{2}}$*is given by*

$$\begin{aligned} \bar{F}({{G}_{1}}\wedge {{G}_{2}})&= {} {{n}_{2}}^{2}\left( 6{{m}_{2}}-{{n}_{2}}^{2}\right) F({{G}_{1}})+{{n}_{1}}^{2}\left( 6{{m}_{1}}-{{n}_{1}}^{2}\right) F({{G}_{2}})+F({{G}_{1}})F({{G}_{2}})\\&\quad -\,3{{n}_{2}}F({{G}_{1}}){{M}_{1}}({{G}_{2}})+{{n}_{2}}\{({{n}_{1}}{{n}_{2}}-1)({{n}_{1}}{{n}_{2}}-4{{m}_{2}})-6{{n}_{1}}{{n}_{2}}{{m}_{2}}\}{{M}_{1}}({{G}_{1}})\\&\quad +\,{{n}_{1}}\left\{ ({{n}_{1}}{{n}_{2}}-1)({{n}_{1}}{{n}_{2}}-4{{m}_{1}})-6{{n}_{1}}{{n}_{2}}{{m}_{1}}\right\} {{M}_{1}}({{G}_{2}})-3{{n}_{1}}F({{G}_{2}}){{M}_{1}}({{G}_{1}})\\&\quad +\,(7{{n}_{1}}{{n}_{2}}-1){{M}_{1}}({{G}_{1}}){{M}_{1}}({{G}_{2}})+8{{n}_{1}}{{n}_{2}}{{m}_{1}}{{m}_{2}}({{n}_{1}}{{n}_{2}}-1).\\ \end{aligned}$$

The proof of the above proposition follows from Proposition 3 with the relevant results from Khalifeh et al. (2009) and De et al.

### Symmetric difference

The symmetric difference of two graphs ${{G}_{1}}$ and ${{G}_{2}}$ is denoted by ${{G}_{1}}\oplus {{G}_{2}}$, so that $|V({{G}_{1}}\oplus {{G}_{2}})|=|V({{G}_{1}})|\times |V({{G}_{2}})|$ and

$$E({{G}_{1}}\oplus {{G}_{2}})=\left\{ ({{u}_{1}},{{u}_{2}})({{v}_{1}},{{v}_{2}}):{{u}_{1}}{{v}_{1}}\in E({{G}_{1}})\quad \text{ or }\quad {{u}_{2}}{{v}_{2}}\in E({{G}_{2}})\; \text{ but } \text{ not } \text{both}\right\} .$$

From definition of symmetric difference it is clear that

$${{d}_{{{G}_{1}}\oplus {{G}_{2}}}}({{v}_{1}},{{v}_{2}})={{n}_{2}}{{d}_{{{G}_{1}}}}({{v}_{1}})+{{n}_{1}}{{d}_{{{G}_{2}}}}({{v}_{2}})-2{{d}_{{{G}_{1}}}}({{v}_{1}}){{d}_{{{G}_{2}}}}({{v}_{2}}).$$

In the following proposition we obtain the F-coindex of the symmetric difference of two graphs.

#### **Proposition 14**

*The F-coindex of*${{G}_{1}}\oplus {{G}_{2}}$*is given by*

$$\begin{aligned} \bar{F}({{G}_{1}}\oplus {{G}_{2}})&= {} {{n}_{2}}^{2}\left( 12{{m}_{2}}-{{n}_{2}}^{2}\right) F({{G}_{1}})+{{n}_{1}}^{2}\left( 12{{m}_{1}}-{{n}_{1}}^{2}\right) F({{G}_{2}})+8F({{G}_{1}})F({{G}_{2}})\\&\quad -12{{n}_{2}}F({{G}_{1}}){{M}_{1}}({{G}_{2}})+{{n}_{2}}\{({{n}_{1}}{{n}_{2}}-1)({{n}_{1}}{{n}_{2}}-8{{m}_{2}})-6{{n}_{1}}{{n}_{2}}{{m}_{2}}\}{{M}_{1}}({{G}_{1}})\\&\quad +{{n}_{1}}\{({{n}_{1}}{{n}_{2}}-1)({{n}_{1}}{{n}_{2}}-8{{m}_{1}})-6{{n}_{1}}{{n}_{2}}{{m}_{1}}\}{{M}_{1}}({{G}_{2}})-12{{n}_{1}}F({{G}_{2}}){{M}_{1}}({{G}_{1}})\\&\quad +4(4{{n}_{1}}{{n}_{2}}-1){{M}_{1}}({{G}_{1}}){{M}_{1}}({{G}_{2}})+8{{n}_{1}}{{n}_{2}}{{m}_{1}}{{m}_{2}}({{n}_{1}}{{n}_{2}}-1). \end{aligned}$$

## Conclusion

In this paper, we have studied the F-coindex of different graph operations and also apply our results to find F-coindex of some special and chemically interesting graphs. However, there are still many other graph operations and special classes of graphs which are not covered here. So, for further research, F-coindex of various other graph operations and composite graphs can be considered.
